# Mucinous Carcinoma of the Breast: A Case Report

**DOI:** 10.7759/cureus.56515

**Published:** 2024-03-19

**Authors:** Jayashree Rey, Samarth Shukla, Sourya Acharya, Pravin Gadkari, Sapna Sihag

**Affiliations:** 1 Pathology, Jawaharlal Nehru Medical College, Datta Meghe Institute of Higher Education and Research, Wardha, IND; 2 Medicine, Jawaharlal Nehru Medical College, Datta Meghe Institute of Higher Education and Research, Wardha, IND; 3 Biochemistry, Dr. Sampurnanand Medical College, Jodhpur, IND

**Keywords:** mucinous carcinoma of breast, treatment, multidisciplinary approach, histopathology, diagnosis, breast cancer

## Abstract

This case report presents the diagnostic journey of a 65-year-old female presenting with symptoms suggestive of breast pathology, ultimately diagnosed with mucinous carcinoma, following comprehensive clinical evaluation and histopathological confirmation. Initial assessments indicated a fibroadenoma; however, subsequent histopathological examination revealed mucinous carcinoma, highlighting the importance of histopathological confirmation in establishing definitive diagnoses. The case underscores the challenges in distinguishing between benign and malignant breast lesions based on clinical presentation and imaging findings alone. The multidisciplinary approach facilitated discussions regarding treatment options tailored to the patient's clinical and pathological characteristics. This case emphasizes the significance of a comprehensive diagnostic approach, integrating clinical evaluation, imaging studies, and histopathological analysis, in ensuring accurate diagnosis and guiding optimal management strategies for patients with breast cancer.

## Introduction

Breast cancer is a significant global health concern, affecting millions of women worldwide. Among the various histological subtypes of breast cancer, mucinous carcinoma represents the rare form of breast carcinoma, comprising approximately 2% of all the diagnosed cases of breast cancer [1]. Mucinous breast cancer carries a relatively favorable prognosis with a low recurrence rate and a low incidence of lymph node metastasis [2]. Diagnostic evaluation of breast lesions typically involves a combination of clinical assessment, imaging studies, and histopathological analysis. Ultrasonography (USG) and computed tomography (CT) scans are commonly utilized imaging modalities to assess the size, location, and characteristics of breast lesions [3]. While USG provides high-resolution imaging, CT scans offer detailed anatomical information, aiding in determining tumor extent and involvement of adjacent structures [4].

Histopathological examination remains the gold standard for definitive diagnosis of breast lesions, including mucinous carcinoma. Biopsy specimens obtained through various techniques, such as fine-needle aspiration (FNA) or core needle biopsy, undergo thorough microscopic evaluation to identify cellular abnormalities indicative of malignancy [5]. The Breast Imaging Reporting and Data System (BIRADS) classification system is widely used to standardize reporting and guide management decisions based on imaging findings [6]. Mucinous carcinoma treatment strategies encompass a multidisciplinary approach, incorporating surgery, chemotherapy, radiation therapy, and targeted therapies tailored to individual patient characteristics and disease stages [7]. Early detection and prompt intervention are crucial in improving outcomes and reducing mortality rates, if any, associated with mucinous carcinoma [8].

## Case presentation

A 65-year-old woman presented to the outpatient department of a specialized hospital with complaints of left breast pain, enlargement, and swelling persisting for two months. She had sought medical attention previously at a local hospital, where she underwent investigations revealing elevated C-reactive protein (CRP) and leukocyte count. Following this, her primary physician referred her to our institution for further evaluation. Upon examination, noticeable enlargement, tenderness, and swelling of the left breast were observed, with no discharge. USG revealed an ill-defined heterogeneously hypoechoic lesion with internal vascularity at the 12 o'clock position in the left breast parenchyma, measuring approximately 3.8 x 1 cm, raising concerns for a neoplastic etiology. Notably, there were no signs of lymphadenopathy.

**Figure 1:**
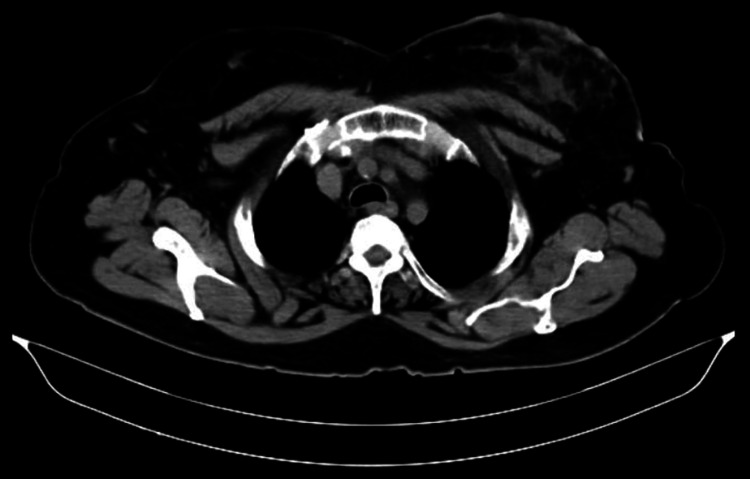
A contrast-enhanced CT scan of the abdomen and pelvis was performed, demonstrating an irregular non-enhancing lesion in the left breast measuring 8.3 x 5.3 x 3.2 cm.

Considering the findings, a CT scan was recommended for a definitive diagnosis. Subsequently, a contrast-enhanced CT (CECT) scan of the abdomen and pelvis was performed, demonstrating an irregular non-enhancing lesion in the left breast measuring 8.3 x 5.3 x 3.2 cm (Figure 1), involving the skin and retroareolar region. The lesion did not extend to the pectoralis major muscle and showed no evidence of necrosis or calcifications suggestive of malignancy. Post-biopsy changes were noted, along with subtle enhancement in the bilateral axillary region, more pronounced on the left side. Additionally, degenerative changes were observed in the visualized spine.

**Figure 2:**
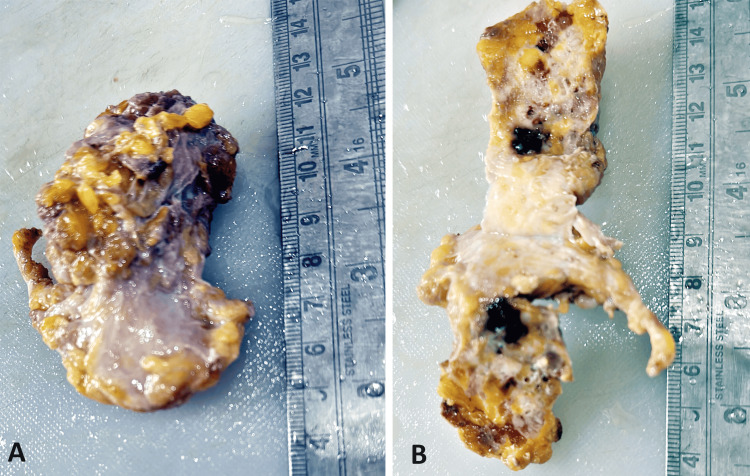
Irregular, greyish-white fibro fatty tissue piece having a soft glistening appearance.

**Figure 3:**
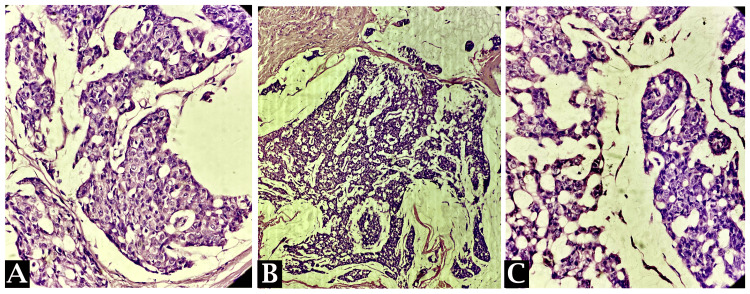
Clusters/nests of tumor cells floating in pools of extracellular mucin. An intermediate nuclear grade in tumor cell clusters is seen.

Following the imaging studies, a USG-guided color Doppler was conducted, and a biopsy of the breast lesion was obtained. Despite initial suspicion of a fibroadenoma, the gross analysis revealed irregular, greyish-yellow fibrofatty tissue with areas of hemorrhage (Figure 2). Further histopathological examination showed features consistent with mucinous carcinoma of the breast (Figure 3). In light of these findings, the patient was diagnosed with mucinous carcinoma of the left breast.

## Discussion

The presented case emphasizes several important clinical and diagnostic considerations in the evaluation of breast lesions, particularly the challenges associated with accurately diagnosing mucinous carcinoma of the breast amidst overlapping clinical and imaging findings. This case underscores the importance of thorough evaluation, including histopathological examination, in accurately diagnosing breast lesions, especially when initial imaging suggests benign pathology. Prompt diagnosis and intervention are critical for optimizing patient outcomes in such cases.

The initial presentation of the 65-year-old female with complaints of pain, breast enlargement, and swelling highlights common symptoms associated with breast pathology, warranting further investigation [9]. In the diagnostic workup, USG and CT scans played pivotal roles in characterizing the lesion, with USG revealing an ill-defined hypoechoic lesion suggestive of a neoplastic etiology [10]. Subsequent CECT provided additional information regarding the extent of the lesion and its involvement with adjacent structures, aiding in the differential diagnosis and treatment planning [11].

Histopathological examination following USG-guided biopsy ultimately confirmed the diagnosis of mucinous carcinoma of the breast, underscoring the importance of tissue analysis in establishing definitive diagnoses [12]. The discrepancy between the initial biopsy findings suggestive of a fibroadenoma and the final histopathological diagnosis of mucinous carcinoma of the breast highlights the inherent limitations of relying solely on imaging and clinical assessments in diagnosing breast lesions [13]. Such discrepancies emphasize the necessity of corroborating imaging findings with histopathological confirmation to guide appropriate management decisions.

The management of mucinous carcinoma typically involves a multidisciplinary approach, integrating surgical resection, chemotherapy, radiation therapy, and targeted therapies based on the tumor characteristics and disease stage [14]. In this case, the diagnosis of mucinous carcinoma prompted discussions regarding treatment options and formulating an individualized treatment plan tailored to the patient's clinical presentation and pathological findings. While the prognosis and long-term outcomes of the patient remain to be determined based on the response to therapy and disease surveillance, the timely diagnosis and initiation of treatment are crucial for optimizing outcomes in patients with mucinous carcinoma [8].

## Conclusions

In conclusion, the presented case of a 65-year-old female underscores the diagnostic challenges inherent in discerning between benign and malignant breast lesions. Despite initial clinical and imaging assessments suggesting a fibroadenoma, subsequent histopathological examination revealed mucinous carcinoma of the breast. This highlights the critical role of histopathological confirmation in establishing definitive diagnoses and guiding appropriate treatment strategies. The multidisciplinary approach facilitated discussions regarding treatment options tailored to the patient's clinical and pathological characteristics. Timely diagnosis and initiation of treatment are paramount in optimizing outcomes for patients with breast cancer. This case emphasizes the importance of a comprehensive diagnostic approach, integrating clinical evaluation, imaging studies, and histopathological analysis to ensure accurate diagnosis and guide optimal management strategies for patients with breast cancer.
